# Isotropic three-dimensional cardiac cine imaging at 0.55T using stack-of-spiral sampling and four-dimensional iterative motion compensation

**DOI:** 10.1016/j.jocmr.2026.102698

**Published:** 2026-01-23

**Authors:** Rajiv Ramasawmy, Ahsan Javed, Daniel A. Herzka, Prakash Kumar, Krishna S. Nayak, Robert J. Lederman, Adrienne E. Campbell-Washburn

**Affiliations:** a Laboratory of Imaging Technology, Cardiovascular Branch, National Heart Lung and Blood Institute, National Institutes of Health, Bethesda, Maryland, USA; b Department of Radiology, Case Western Reserve University and University Hospitals, Cleveland Medical Center, Cleveland, Ohio, USA; c Ming Hsieh Department of Electrical and Computer Engineering, University of Southern California, Los Angeles, California, USA; d Laboratory of Cardiovascular Interventions, National Heart Lung and Blood Institute, National Institutes of Health, Bethesda, Maryland, USA

**Keywords:** Three-dimensional cine, Stack-of-spiral, Free-breathing, Isotropic, Motion-compensated reconstruction, 0.55T MRI

## Abstract

**Background:**

Isotropic three-dimensional (3D) cine imaging is an attractive one-stop-shop acquisition for cardiac MRI, as it can be arbitrarily resliced for the assessment of cardiac function and simplifies imaging workflows. Current free-breathing 3D cine approaches are hampered by long reconstruction times, and at lower-field strengths, by relatively long acquisition times. Here, we aim to maximize acquisition efficiency at 0.55T pairing two techniques; using a spiral acquisition with an optimized sampling distribution and a reconstruction incorporating data from all respiratory phases.

**Methods:**

We implemented a 2 mm isotropic 3D cine approach on a prototype 0.55T scanner, using a 6 min stack-of-spiral balanced steady-state free precession (bSSFP) acquisition modified to use tiny-golden-angle in-plane rotations and distribute the k_z_ partition samples to a variable-density. The data were reconstructed with a modified iterative motion compensation reconstruction which resolved cardiac motion (denoted 4D iMoCo) and combined respiratory states using a navigator signal extracted from the acquired data. The proposed technique was compared to reference 2D free-breathing Cartesian volumetry of the left ventricle in 11 human subjects.

**Results:**

The 4D iMoCo reconstruction required 20 min. The proposed variable-density sampling distribution reduced image artifacts, compared to a common linear sampling approach, and improved apparent signal-to-noise with relative increase of 221 ± 99%. Measurements had good agreement with the 2D Cartesian reference data with a left-ventricular volume bias of −2.5 ± 6.2% and 2.6 ± 10.4% in diastole and systole, respectively, and an ejection fraction bias of −3.5 ± 8.8%.

**Conclusion:**

We demonstrate an efficient free-breathing technique to produce 2 mm isotropic 3D cardiac images within a 6 min acquisition time and 20 min reconstruction time at 0.55T. Such a method could be a valuable clinical tool for cardiac imaging.

## Graphical abstract

**Figure:**
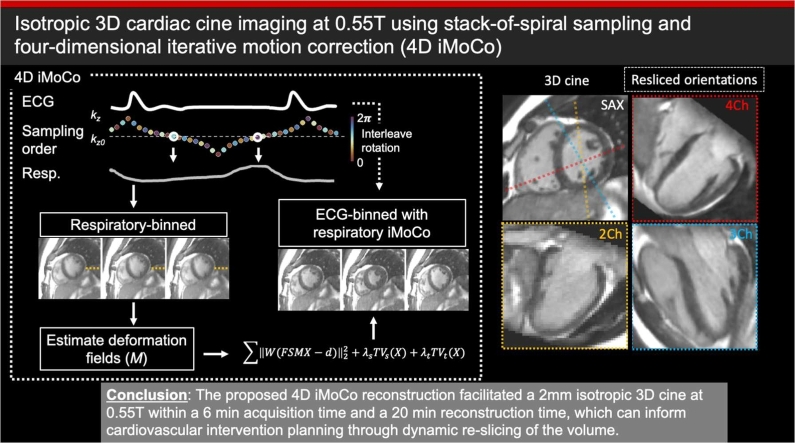

## 1 Introduction

Three-dimensional (3D) cardiac cine imaging is a patient-friendly alternative to traditional multi-slice two-dimensional (2D) [1], [2], [3], [4]. These free-breathing, isotropic acquisitions can be resliced into arbitrary orientations to simplify imaging [5]. Typically, 3D radial trajectories with interleaved navigator readouts are favored and “5D” reconstructions have been demonstrated to resolve both cardiac and respiratory motion.

Recently, an iterative motion corrected (iMoCo) image reconstruction approach was demonstrated for pulmonary imaging to combine all respiratory phases and achieve 100% acquisition efficiency [6]. This approach accounts for breathing motion via an intermediate respiratory-resolved reconstruction and incorporates the deformation fields to generate a single higher-quality image from all motion states.

In this study, we demonstrate a 6-minute free-breathing 3D cine at 0.55T with 2 mm isotropic resolution, using a stack-of-spirals for improved signal-to-noise (SNR) efficiency with an optimized sampling pattern in the slice dimension. We extend the iMoCo technique to add a cardiac dimension, such that data from all respiratory states can help drive the reconstruction to produce a 3D cardiac-resolved cine.

## 2 Methods

### 2.1 Ethics statement

We obtained institutional review board approval and written informed consent for imaging healthy volunteers (n = 9) and two patients with cardiac disease (one with non-ischemic cardiomyopathy and one with left-ventricular non-compaction cardiomyopathy). The subjects were 7/4 Male/Female, with a mean age of 32 years (range 23–44 years) and a mean body mass index (BMI) of 26.5 kg/m^2^ (range 19.7–40.9 kg/m^2^).

### 2.2 4D iMoCo reconstruction

**Fig. 1:**
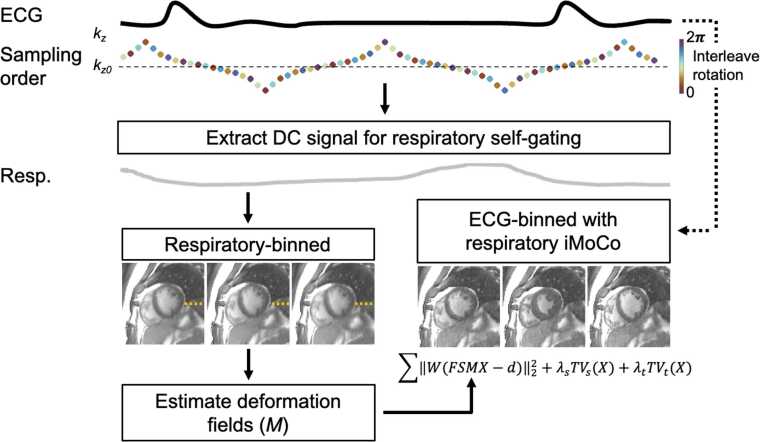
Schematic of the 4D iterative motion-compensation reconstruction, using a stack-of-spiral acquisition with a variable-density distribution of slice sampling and a golden-angle spiral rotation between successive TRs. The respiratory signal (Resp.) is extracted from the center of k-space samples (k_0_, “DC signal”) acquired every 360 ms, to bin the data for cardiac-averaged, respiratory-resolved image reconstruction. Motion fields (M) are estimated using image registration and used within the 4D iMoCo reconstruction to produce cardiac-resolved images with 100% of the data. *4D* four-dimensional, *TR* repetition time, *iMoCo* iterative motion-compensation reconstruction

We expanded the iMoCo technique to a 3D+time reconstruction, with an additional cardiac dimension (Fig. 1), within the Gadgetron framework [7]. Reconstructions were performed on a system equipped with Dual AMD EPYC processors (Advanced Micro Devices, Inc, Santa Clara, California), 1 TB RAM, 4x Nvidia A100 GPUs (NVIDIA, Santa Clara, California).

The reconstruction has two steps as follows: 1) an intermediate respiratory-resolved, cardiac-averaged image reconstruction, and 2) a respiratory-motion-compensated, cardiac-resolved reconstruction.

ECG was used for cardiac gating and respiratory navigation used “DC” signal from the center of k-space (kx, ky, and kz = 0) sampled every 360 ms, using all the channel data. The data were binned into eight cardiac-averaged respiratory phases and respiratory-resolved images were reconstructed using a spatio-temporal total-variation (TV) reconstruction [7].

Deformation fields were estimated from the respiratory resolved images registered to the end-expiratory state using an optical flow-based method [8]. Deformation fields were then included in the iterative motion-corrected reconstruction to reconstruct 25 cardiac phases with data from all respiratory positions using the following cost function:

$$\mathrm{argmi}n_{X}\sum_{n,k,c}^{N_{c}\mathrm{KC}}{\left|\left|W_{k,c}\left(F{S_{n}M}_{k}X_{c}-d_{n,k,c}\right)\right|\right|}_{2}^{2}+\lambda_{s}TV_{s}\left(X\right)+\lambda_{t}TV_{t}(X)$$

where *d*_*nkc*_ is motion-resolved multi-channel data, *X* is the end-expiration cardiac-resolved images to be reconstructed, *M*_*k*_ is estimated motion field across respiratory positions, *S*_*n*_ is coil-sensitivity of the nth channel, *F* is the non-uniform Fourier transform, *W*_*k*_ is the density compensation weight for each respiratory and cardiac motion states, and TV represents the spatial (*TV*_s_) and temporal (*TV*_t_) total variation terms with the associated Lagrange multipliers (*λ*). *N*_*c*_ represents total number of channels, *K* is the total number of respiratory states, and *C* is the total number of cardiac phases. The following parameters were selected following visual assessment across three subjects: *λ*_s_ = 0.05, *λ*_*t*_ = 5, and 15 iterations, using the first 12 channels following principle-component analysis. 4D iMoCo reconstruction code with an example dataset can be found at https://github.com/NHLBI/lit_gadgetron/tree/cardiac_4D_imoco. We compared the 4D iMoCo cine reconstruction to respiratory averaged and end-expiration images generated using 40% of the data from the most stable respiratory position.

### 2.3 Data sampling optimization

We experimentally optimized an equispaced stack-of-spirals slice (k_z_) sampling to minimize large gaps around the center of k_r_-k_z_ space after retrospective cardiac-binning. We designed a k_z_ acquisition order with variable density approximating a Gaussian distribution, and we tested a golden-angle rotation per TR.

**Fig. 2:**
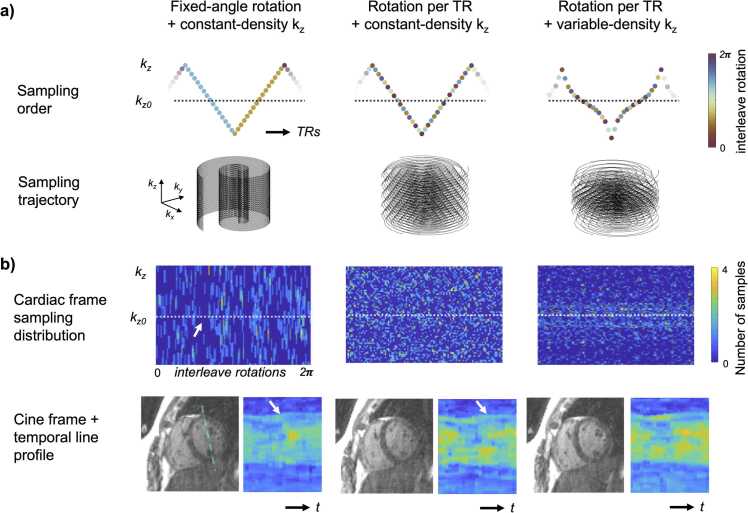
Comparison of 3D acquisition ordering: a) The sampling order plots the slice-encoding on the y-axis and represents the spiral interleave angular rotation in color. The sampling trajectory below depicts the three-dimensional k-space filling for 80 consecutive TRs. b) The golden-angle per TR and constant-density kz sampling will yield a more randomly distributed k-space filling per cardiac phase. The variable-density *k*_z_ sampling order acquires more data around the center of k-space (dashed line), and as a result, avoided large gaps near the k-space center (arrow), improving image contrast and observed aliasing—which can be appreciated from the improved conspicuity of the papillary temporal line profiles (arrow). *3D* three-dimensional, *TR* repetition time

We compared three sampling approaches as follows: 1) fixed-angle per slice-loop (followed by a rotation) with constant density k_z_ sampling, 2) rotation per TR with constant density k_z_ sampling, and 3) rotation per TR with a variable-density distribution in k_z_ (Fig. 2a). The rotation angle was a pseudo-tiny-golden-angle (approximately ψ_4_: 39.3°) [9].

### 2.4 Imaging protocols

Imaging data were acquired on a 0.55T system (prototype MAGNETOM Aera, Siemens, Erlangen, Germany) with a maximum gradient amplitude and slew rate of 43 mT/m and 180 mT/m/ms per axis, using a 6-channel chest coil and 12-channels of a spine coil. Free-breathing bSSFP 3D stack-of-spirals acquisitions (2.0 mm isotropic, TE/TR=1.1/4.5 ms, minimum phase RF with volume-selective excitation, flip-angle = 50°, 64 partitions with 25% over-sampling, total acquisition time 5 min 50 s). We used a short-axis volume prescription, as this was most efficient to cover the left-ventricle with a slab-selective pulse. Spiral readouts were designed using a variable density trajectory [10], with a 32-shot design, 384×384 mm^2^ field-of-view with a 192×192 matrix, 119 rotations, and 2.5 ms readout duration. To minimize eddy currents and concomitant fields, the gradient amplitude was limited to 16 mT/m.

For left-ventricular volumetry, we compared our 3D acquisition to reference free-breathing 2D bSSFP cine stack (1.9×2.5×8.0 mm^3^, TE/TR = 1.3/3.2 ms, flip-angle = 80°, 11 slices with 25% gap, total acquisition time ∼3.5 min), reconstructed with a real-time motion compensation [11].

### 2.5 Quantitative analysis

2D and 3D cine datasets were semi-automatically segmented by an operator with 9 years of cardiac imaging in SuiteHeart (NeoSoft, Pewaukee, Wisconsin, USA) to compare left-ventricular end-systolic volume (LV-ESV), end-diastolic volume (LV-EDV), and ejection fraction (EF), using Bland-Altman and Wilcoxon matched pairs analysis for statistical significance.

To compare image reconstruction approaches, blood-myocardium edge sharpness was measured following a sigmoid curve fit using three line profiles in a diastolic mid-short-axis slice [12]. Apparent signal-to-noise-ratio (aSNR) was measured as a proxy estimate of signal-to-noise and sampling artifacts within the left-ventricular blood pool and myocardium (with mean region-of-interest surface areas 14.4 ± 2.4 and 12.8 ± 2.3 cm^2^, respectively) using the median signal within a region-of-interest divided by the standard deviation of a noise-region drawn outside of the body (with surface areas ranging from 26–36 cm^2^), averaged across the cardiac cycle, and compared between reconstruction approaches.

Acquisition ordering schemes were compared using aSNR and frequency of sampling at the center of k-space, using Friedman tests. The apparent contrast-to-noise-ratio (aCNR) between blood and myocardium was also compared between sampling schemes and the Cartesian reference.

## 3 Results

### 3.1 Sampling scheme

The isotropic 3D cine acquisition had an average temporal resolution of 39.1 ± 5.7 ms. The images exhibited reduced under-sampling artifacts using the variable-density slice sampling scheme (Fig. 2b), due to the smaller holes at the center of k_z_-k_r_ space. The percentage acquired samples at the central slice partition (k_z_=0) across cardiac frames (Table 1) showed a significant increase for the golden-angle and variable-density sampling approach (28% vs 62%, p < 0.01 using a Friedman test). The golden-angle and variable-density sampling showed a significant (p < 0.01) increased blood aSNR and aCNR versus the constant kz sampling schemes. The aCNR for the variable-density sampling was 11 ± 3% compared to the 2D Cartesian acquisition.

**Table 1:** Comparison of sampling approaches and resulting average percentage filling of the central partition (*k*_z_=0) per cardiac phase, resulting in improved apparent SNR and CNR metrics for the golden-angle rotation and variable density spiral acquisition following 4D iMoCo reconstruction, though these values were lower than the reference 2D Cartesian acquisition

	Cartesian	Fixed-angle rotation + constant-density k_z_	Rotation per TR + constant- density k_z_	Rotation per TR + variable-density k_z_
Acquired Resolution	1.9 × 2.5 × 8.0 mm^3^	2 mm^3^	2 mm^3^	2 mm^3^
Central partition % fill		27.9±3.8%	27.6±4.2%	62.0±6.0%*
Blood aSNR	206±107	17±13	23±13	30±15*
Myocardial aSNR	62±31	9±8	12±8	15±8*
aCNR	144±77	8±6	10±6	15±8

*SNR* signal-to-noise ratio*, CNR* contrast-to-noise ratio*, 2D* two-dimensional*, 4D* four-dimensional*, iMoCo* iterative motion-compensation reconstruction*, TR* repetition time*.* Averages and standard deviations are reported across six normal subjects, significant values have been denoted with a star

### 3.2 4D iMoCo reconstruction

**Fig. 3:**
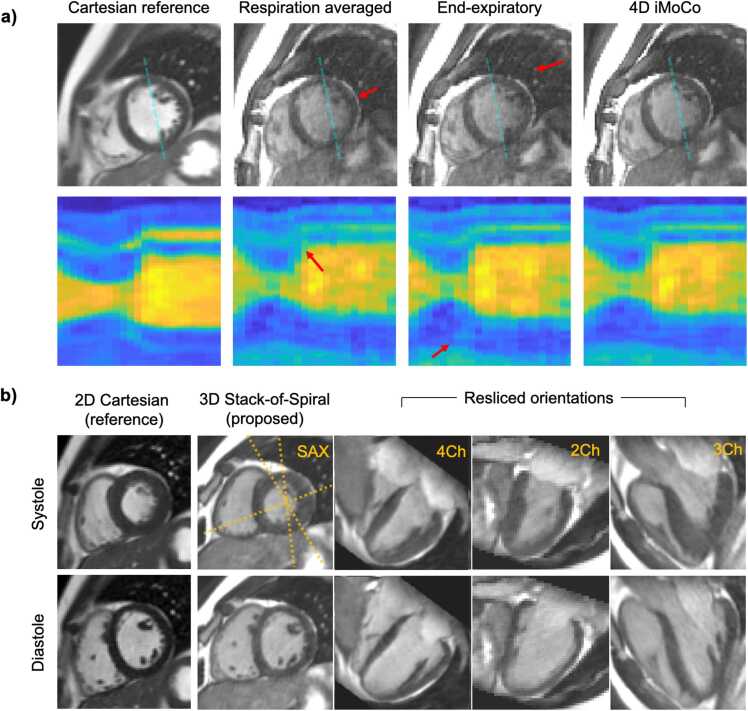
a) Comparison of 2D Cartesian reference, and 3D spiral cine with respiration averaged, end-expiratory, and cardiac-resolved iMoCo reconstructions (left-to-right) in a healthy human subject. Spiral cines with respiration iMoCo demonstrate improved myocardium-blood pool contrast sharpness and reduced aliasing over averaged reconstructions and stable binning (arrows) and compare well with reference acquisitions for structure and contrast. b) Example images showing the isotropic 3D cines against a reference 2D Cartesian acquisition in systole and diastole and demonstrating re-slicing the three-dimensional image from the original SAX orientation to typical cardiac planes: 4Ch, 2Ch, and 3Ch views (dashed guidelines indicating the resliced orientations). In this example, the 2Ch was the most oblique orientation from the acquired short-axis, yielding some pixelation in the interpolation and a slice thickness of 3.2 mm. *2D* two-dimensional, *3D* three-dimensional, *iMoCo* iterative motion-compensation reconstruction, *SAX* short-axis, *4Ch* four chamber, *2Ch* two chamber, *3Ch* three-chamber

The 4D iMoCo reconstruction improved image blurring and artifacts compared to respiratory-averaged and end-expiratory images (Fig. 3a and **Supplemental Video 1**). Sharpness showed a non-significant improvement (Friedman test, p > 0.05) comparing respiratory averaged, end-expiratory, and iMoCo reconstructions (distance of 2.4 ± 1.6, 2.3 ± 1.4, 1.9 ± 1.2 pixels, respectively).

The total 4D iMoCo reconstruction time (in 3 subjects) was 20.1 ± 1.8 min (4.7 ± 0.6 min for data loading, cardiac-binning, and respiratory self-gating; 3.5 ± 0.1 min for respiratory-resolved reconstruction; and 10.8 ± 0.2 min for 4D iMoCo).

### 3.3 Isotropic image quality

Isotropic 3D cine provided good blood-myocardium contrast, including when resliced to typical cardiac planes (Fig. 3b**)**. Fine details such as the papillary muscles and valves are well-visualized in the 3D cine images (Supplemental Video 2). Supplemental Video 3 demonstrates good-quality imaging across a range of body types and physiology patterns from five subjects (four healthy volunteers and one patient).

### 3.4 Cardiac function assessment and qualitative comparisons

The mean LV-ESV bias between the reference 2D and the 3D cine was −1.3 ± 7.5 mL, the mean LV-EDV bias was 3.8 ± 4.5 mL and the mean EF bias was 2.3 ± 5.1%. No significant difference between reference 2D and 3D cines was found for each volumetric measurement (Wilcoxon matched pairs, p > 0.05). Bland-Altman plots are provided in Supplemental Figure 4.

## 4 Discussion

In this study, we optimized a 3D stack-of-spirals acquisition combined with a 4D iMoCo reconstruction to produce a 2 mm isotropic cine with a non-contrast 6-minute acquisition and 20-minute reconstruction time at 0.55T. The variable-density in k_z_ improved reconstruction quality by minimizing large gaps around the center of k-space, and the novel implementation of a 4D iMoCo reconstruction used 100% sampling efficiency to reduce artifacts and improve SNR.

### 4.1 Acquisition ordering, self-gating & sampling approaches

The variable density sampling in k_z_ reduced artifacts after 4D iMoCo reconstruction compared to linear slice-ordering. The variable-density sampling did not produce any perceivable increase in artifacts due to eddy currents, potentially due to small k_z_-steps between successive TRs.

The DC signal self-gating was successful in all subjects tested in this study, though a smaller sampling interval will be explored in the future. Free-breathing acquisitions are advantageous for patients who cannot breath-hold, including elderly and pediatric subjects.

Though previous 3D cardiac applications have used fat-suppression techniques to improve image quality, we chose not to disrupt the steady-state and maximize overall acquisition sampling efficiency. The 6-minute scan time is similar to previously demonstrated contrast-free, isotropic acquisitions across field strengths ranging from 2–13 min [1], [2], [13], [14].

### 4.2 4D iMoCo reconstruction

We expanded the iMoCo reconstruction to resolve cardiac motion and correct for respiratory motion during a free-breathing acquisition. In this study, we prioritized SNR-efficiency, and chose to combine the data in to one respiratory phase where previous literature has used 5D reconstructions that resolve both cardiac and breathing motion [1], [2], [14], [15].

We observed some minor residual “streaking” type artifacts, which are common in accelerated non-Cartesian acquisitions. Accelerated imaging can be more difficult at low-field due to the reduced SNR, and increased g-factors due to the larger receive coil elements[16]. In addition, fat signal is very bright due to its short T1 at 0.55T and can lead to relatively high intensity artifacts. However, an advantage of lower-field imaging is reduced off-resonance blurring caused by fat, and as such we determined to limit the readout duration and not apply non-Cartesian deblurring to minimize reconstruction time.

The reconstruction was deployed on GPUs, which enabled reconstruction times of 20 min. This reconstruction time is feasible for images to be returned inline during an exam, in workflows where additional sequences are acquired while a reconstruction task runs. Previous studies have reported 3D cine reconstruction times of 1.5–2.9 h [2], [14]. Newer generation GPUs report an approximate 4× increase in processing speed, which could improve the 4D iMoCo reconstruction time.

### 4.3 Analysis and image quality

Left-ventricle volumetry correlated well with reference 2D imaging with the difference in mean LV-ESV, LV-EDV, and EF less than 5%, though some differences may be due to the temporal regularization in the 4D iMoCo reconstruction. Although breath-held acquisitions are more common, a previously validated free-breathing 2D cine was used in this study [17].

We observed good blood-myocardium contrast in our 3D cine, where previous literature has documented reduced blood-myocardium contrast in 3D bSSFP imaging, due to the reduced inflow of blood into the larger excited region [18]. The CNR values we measured are within the reported ranges for 3D cine. During this study, we observed an orientation dependence of left-ventricular blood signal intensity, such that when excited volumes saturate signal from both lungs (e.g., 4-chamber and 2-chamber planning), the contrast was reduced (Supplemental Fig. 5).

## 5 Limitations

We included a limited range of patients with cardiovascular disease, and robustness needs further evaluation across subjects with higher BMI, structural abnormalities, and devices. Furthermore, right ventricle volumetry was not quantified in our study, due to inconsistent coverage of the whole heart when using a slice-selective short-axis prescription.

Our 4D iMoCo extension did not include off-resonance or concomitant field corrections in the reconstruction [7], as this would increase reconstruction times, but potentially improve image sharpness.

## 6 Conclusion

We demonstrate a 2 mm isotropic free-breathing 3D cine at 0.55T using an optimized stack-of-spiral sampling scheme, and a novel cardiac-resolved iMoCo reconstruction. The 3D cine can be a “one-stop” acquisition for diagnostic volume measurements, ideal for patients with structural abnormalities and pre-procedural planning for intervention by facilitating patient-tailored image reformatting.

## Author contributions

**Rajiv Ramasawmy:** Writing – original draft, Visualization, Software, Methodology, Formal analysis, Data curation, Conceptualization. **Ahsan Javed:** Writing – review & editing, Methodology, Conceptualization. **Daniel A. Herzka:** Writing – review & editing, Conceptualization. **Prakash Kumar:** Writing – review & editing, Methodology. **Krishna S. Nayak:** Writing – review & editing, Methodology. **Robert J. Lederman:** Writing – review & editing, Resources, Conceptualization. **Adrienne E. Campbell-Washburn:** Writing – review & editing, Writing – original draft, Supervision, Resources, Investigation, Conceptualization.

## Declaration of competing interests

The authors declare that they have no known competing financial interests or personal relationships that could have appeared to influence the work reported in this paper.
