# Supplementary material for: Isotropic three-dimensional cardiac cine imaging at 0.55T using stack-of-spiral sampling and four-dimensional iterative motion compensation
Source: J Cardiovasc Magn Reson. 2026 Jan 23;28(1):102698. doi: 10.1016/j.jocmr.2026.102698 (PMC13213869; doi:10.1016/j.jocmr.2026.102698)
Supplement: Supplementary file 1 — Supplementary material [file mmc1.docx]

# Supplemental Materials

## Supplemental Video 1

| 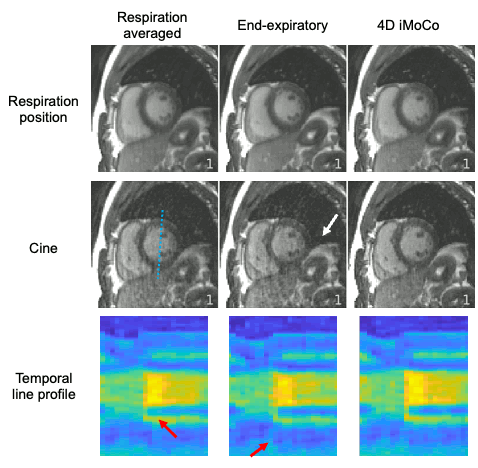 |
| --- |
| Supplemental Figure 1: Example animation demonstrating the respiratory navigation strategies on the final cardiac-resolved cine reconstruction. The top row shows an example of the respiratory position for averaged, end-expiratory and the respiratory-resolved images that will be used for iMoCo reconstruction (left-to-right). The middle row shows the corresponding cardiac-resolved reconstruction using an iterative reconstruction. As expected, an improvement in sharpness can be observed in the end-expiratory images over the respiratory-averaged data (white arrow), and the iMoCo reconstruction further improves the signal-to-noise by using most of the data. Line profiles have also been plotted (dashed line), where the improvement in sharpness and signal-to-noise in the iMoCo can be seen within the heart (red arrows). |

## Supplemental Video 2

| 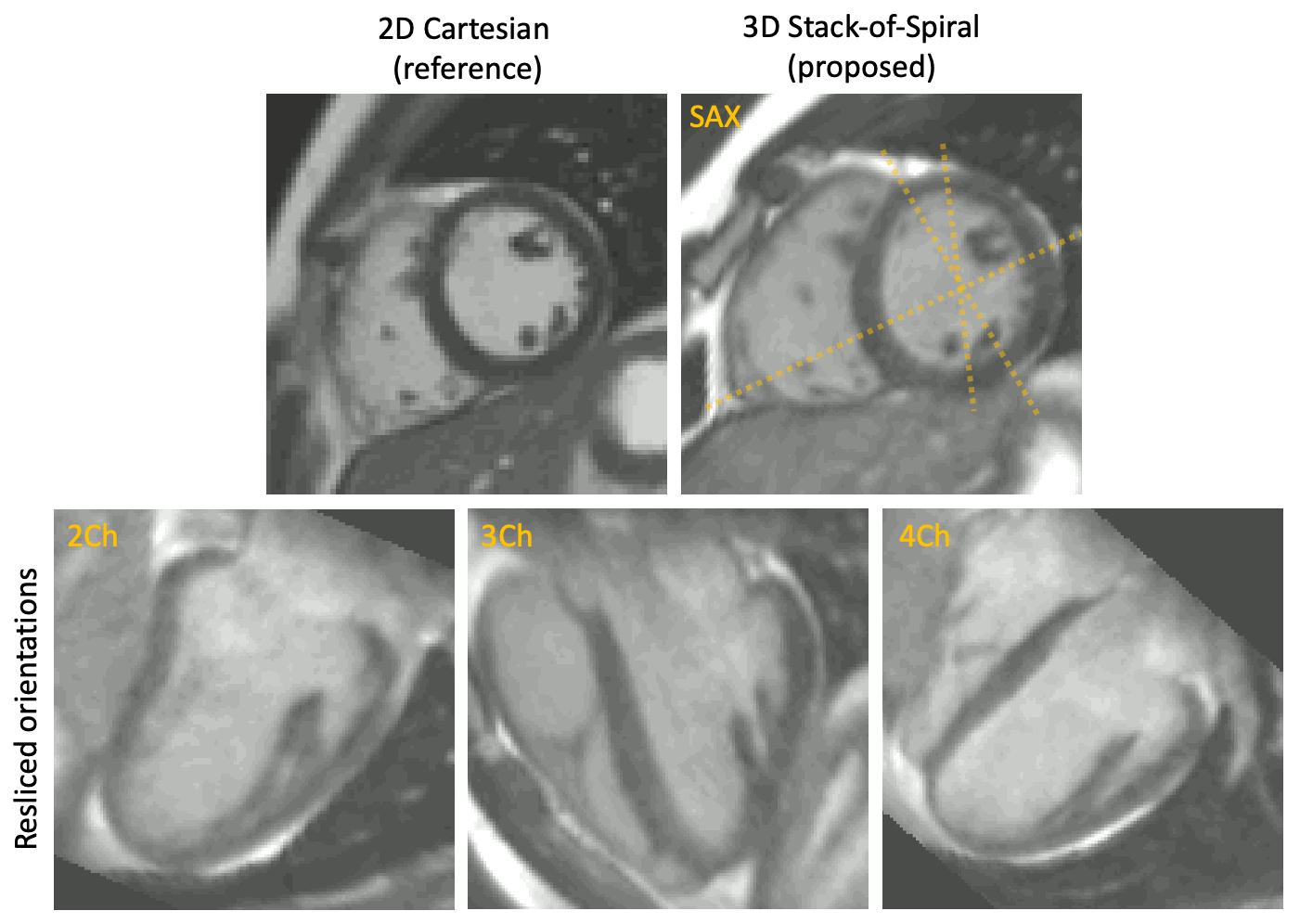 |
| --- |
| Supplemental Figure 2: Example movie demonstrating re-slicing of the 3D cines. The top row has one slice from the 2D Cartesian reference, and the 3D cine acquired in the short axis (SAX) orientation. The bottom row demonstrates three typical cardiac planes: four chamber (4Ch), two chamber (2Ch) and three-chamber (3Ch) views (dashed guidelines indicating the resliced orientations). |

## Supplemental Video 3

| 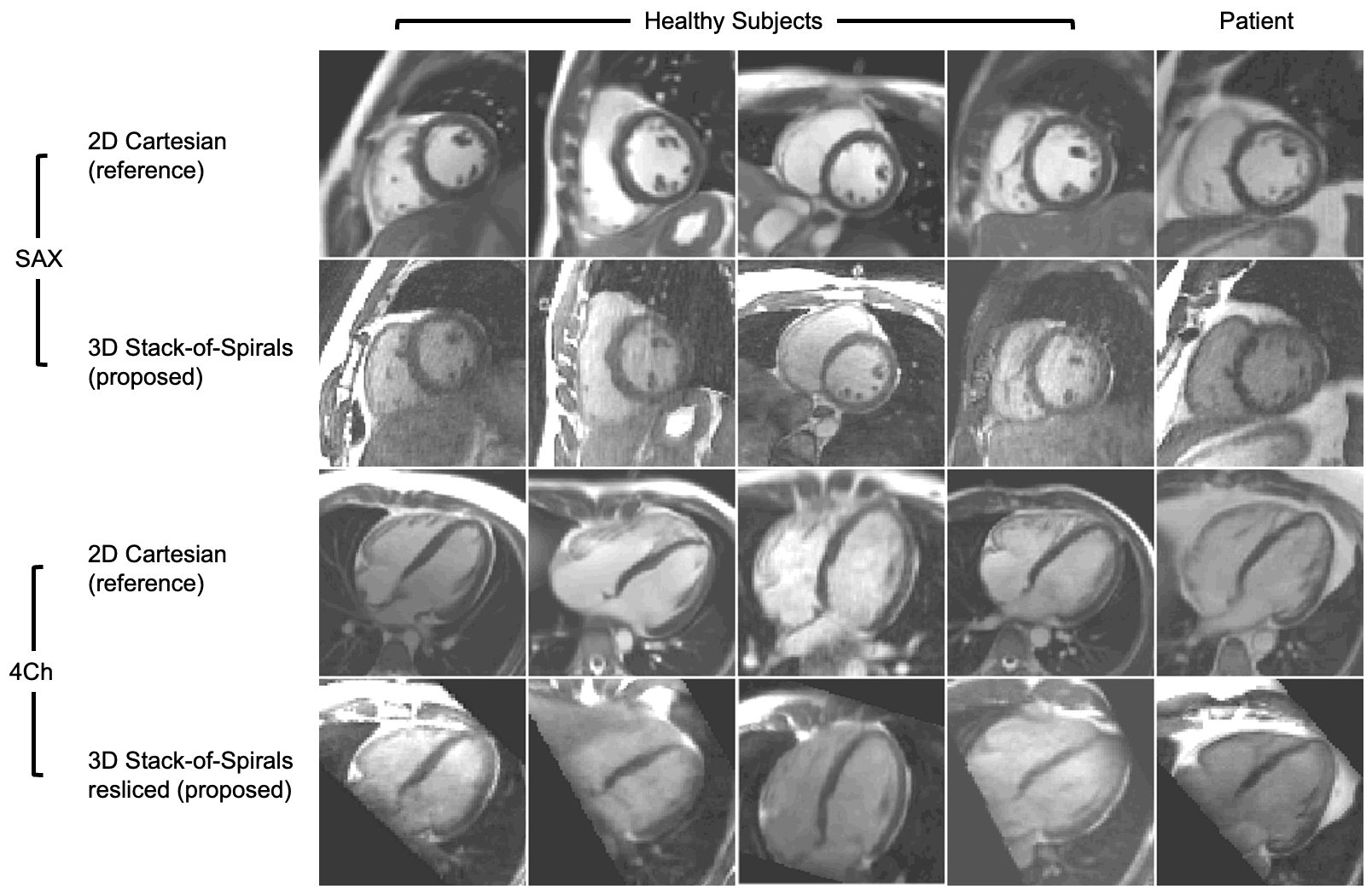 |
| --- |
| Supplemental Figure 3: Animation comparing free-breathing cines across five subjects. The 5^th^ column demonstrates imaging in a patient with left-ventricular non-compaction cardiomyopathy. 2D imaging was sequentially acquired in the short-axis (SAX) and four-chamber (4Ch) orientations, and isotropic 3D imaging acquired in the short-axis and subsequently resliced into a four-chamber view. The isotropic imaging has lower in-plane resolution but can resolve structure in arbitrary resolutions. |

## Supplemental Figure 4

| 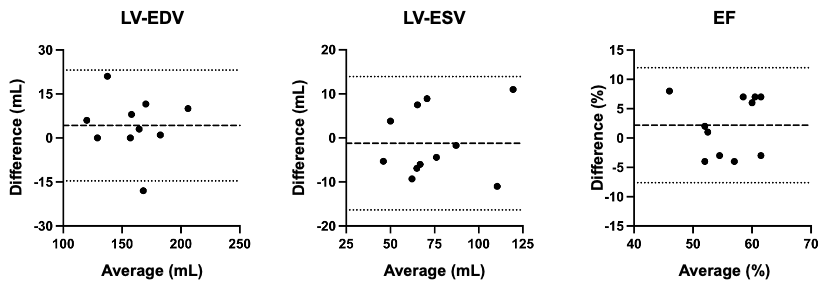 |
| --- |
| Supplemental Figure 4: Bland-Altman analysis for comparing left-ventricular end-systolic volume (**LV-ESV**), end-diastolic volume (**LV-EDV**) and ejection fraction (**EF**) between multi-slice 2D and isotropic 3D cines across 11 subjects. The mean LV-ESV bias was -1.3 ± 7.5 ml, the mean LV-EDV bias was 3.8 ± 4.5 ml, and the mean EF bias was 2.3 ± 5.1%. |

## Supplemental Figure 5

| 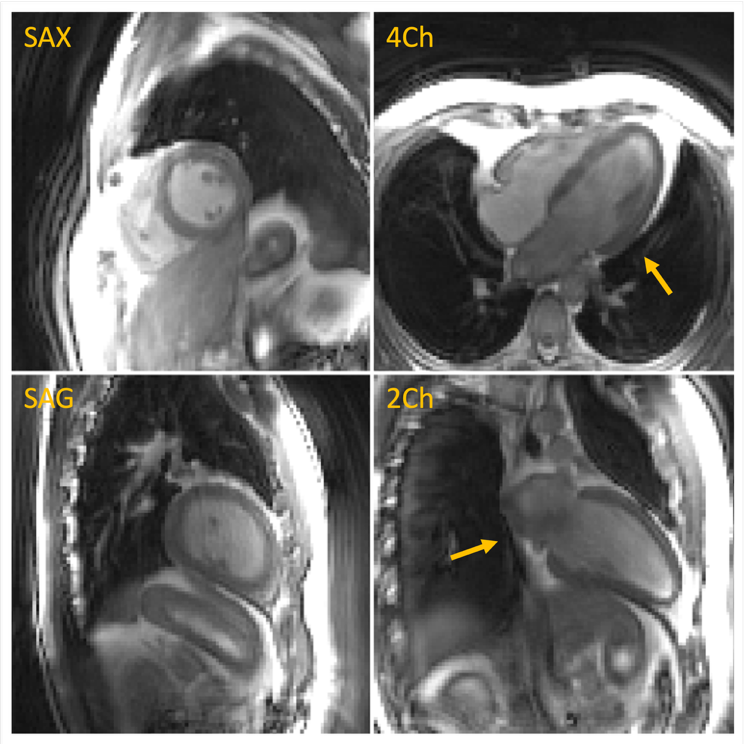 |
| --- |
| Supplemental Figure 5: Orientation dependence on volume left ventricular blood-myocardial contrast acquired in an anisotropic dataset (2x2x8mm^3^) and averaged across the cardiac cycle and respiration. The excitation volume was chosen to match the isotropic volume used in this study. It was observed that excitation volumes that cover the left-ventricle but incidentally excite both lungs, such as the four- and two-chamber views (4Ch, 2Ch), resulted in reduced left ventricular blood pool signal intensity (arrows), whereas the short-axis (SAX) and a sagittal (SAG) orientation demonstrated better contrast due to only one of the lungs not having their signal saturated. The short-axis orientation was chosen for this study for an optimal contrast and coverage of the left ventricle. |
